# Cure of Ague by Iodide of Potassium

**Published:** 1856-07

**Authors:** 


*Cure of Ague by Iodide of Potassium.*—The object of my writing is to state to my professional brethren that I have used the iodide of potassium now in considerably more than a hundred cases, and have never yet failed in curing the disease very quickly. In some cases, where the disease has been of long standing, and the patient very much reduced, I have added a grain or two of quinine to each dose of the iodide of potassium; but my general prescription has been for an adult: ℞ Potas. iodid. ℥iss (?); aquæ menth. pip. ℥xij. M. Coch. mag. ij. 4tâ quâque horâ sumend. So that there could be no doubt what was the remedy that cured the disease. In proof of the value of this drug, I will only mention one case out of all that I have thus treated.

Mrs. Smith, aged 59, sent for me last month, having suffered from tertian ague, off and on, since September. Not being in very good circumstances, she went to the clergyman's wife of the parish in which she resided, who very kindly gave her some quinine, telling her it was no use sending for the medical man, as he must give her the same remedy. However, not getting well, she sent for me. After hearing what I have related, and finding that she had a tolerable pulse, her bowels open, and motions healthy, with a clean tongue, I sent her nothing but the above mixture; and she never had a return of the ague after the second she took of it.—*Mr. Sankey of Beckley, in Association Med. Jour.*, (*in Dublin Med. Press.*)

